# Severe ovarian hyperstimulation syndrome and gonadotropin-releasing hormone agonist trigger in patients with hypogonadotropic hypogonadism: A report of two cases

**DOI:** 10.4274/tjod.galenos.2020.65031

**Published:** 2020-12-10

**Authors:** Ali Sami Gürbüz, Funda Göde, Fatma Kılıç, Zeynep Umay Gürbüz, Rüya Deveer

**Affiliations:** 1 Novafertil IVF Center, Clinict of Obstetrics and Gynecology, Konya Turkey; 2 Bahçeşehir University Faculty of Medicine, Department of Obstetrics and Gynecology, İstanbul, Turkey; 3 Necmettin Erbakan University Faculty of Medicine, Department of Obstetrics and Gynecology, Konya, Turkey; 4 Koç University Faculty of Medicine, İstanbul, Turkey; 5 Sıtkı Koçman University Faculty of Medicine, Department of Obstetrics and Gynecology, Muğla, Turkey

**Keywords:** ART, freeze-all, GnRH agonist trigger, hypogonadotropic hypogonadism, OHSS

## Abstract

Ovarian Hyperstimulation syndrome (OHSS) is a rare condition in patients with hypogonadotropic hypogonadism. Two patients with hypogonadotropic hypogonadism are reported, a rare case of severe OHSS and a case of prevented OHSS via gonadotropin-releasing hormone (GnRH) agonist trigger, respectively. The first case was a 31-year-old patient. In vitro fertilization (IVF) treatment was performed three times but the patient never developed OHSS. The first patient was diagnosed as having severe OHSS on the ninth day after the fresh embryo transfer. She stayed 66 days in hospital and 50.5 litres of fluid were aspirated from her abdomen. The second case was a 26-year-old and primary infertile patient. She had never undergone IVF treatment. The GnRH agonist stimulation test was performed before IVF treatment. After the ovarian stimulation, GnRH agonist trigger was given. Thirty-two oocytes were retrieved from the ovaries and OHSS did not occur. Although severe OHSS is rare, it can develop in patients hypogonadotropic hypogonadism. If a GnRH stimulation test is performed before ovarian stimulation, OHSS can be prevented because the test allows agonist triggering instead of hCG in hypogonadotropic hypogonadism.

## Introduction

Hypogonadotropic hypogonadism (HH) is a puberty-delay and amenorrhea-causing disorder due to deficiency of gonadotropins and sex steroids in the circulation. Individuals with HH have defects either in the secretion of the gonadotropin-releasing hormone (GnRH) from hypothalamus or GnRH receptors in the hypophysis^(1)^. Ovaries are not stimulated depending on the deficiency of gonadotropins, thus, infertility follows in HH. Stimulation with exogen gonadotropins is crucial for fertility treatment because folliculogenesis and ovulation are defective in patients with HH. Human chorionic gonadotropin (hCG) injection is mandatory to mature oocytes in controlled ovarian stimulation because the luteinizing hormone (LH) peak does not exist in these patients^(2)^.

Ovarian Hyperstimulation syndrome (OHSS) is the most serious, life-threatening, iatrogenic complication of assisted reproductive technology (ART) cycles. HCG is the major molecule that causes OHSS^(3)^. Therefore, to prevent and manage OHSS, GnRH agonists have been started to be used for final oocyte maturation. As a result, a segmented approach, including GnRH agonist triggering plus freezing of all embryos was recommended for the complete prevention of OHSS, especially for patients with an extreme ovarian response^(4)^.

In general, OHSS is not common in patients with HH and hCG is the primary choice for oocyte maturation^(5)^. However, in some patients with HH, a hyper response may also occur and hCG triggering might be a life-threatening event in these patients. The exact pathology in patients with HH may be related to pituitary receptors or hypothalamus, and a patient with HH may not respond to GnRH agonists efficiently. Therefore, theoretically, GnRH agonists are not recommended to be used for triggering oocyte maturation in patients with HH^(6)^.

In the following article, HH and agonist trigger will be discussed considering two patients with HH. In the first case, severe OHSS was seen, in the second, potential OHSS development was prevented using a GnRH agonist trigger.

## Case Reports

The patients were treated in Novafertil *In vitro* Fertilization (IVF) Center, Konya, Turkey. Institutional review board approval was given (NIRB:2018-22) and written informed consent was obtained from the patients.

In 2013, a 31-year-old patient presented to our IVF center with symptoms of primary amenorrhea and secondary infertility. She was married for 11 years and had undergone a right salpingo-oophorectomy due to a dermoid cyst. Due to having HH, she underwent intrauterine insemination (IUI) treatment three times with hMG and IVF treatment three times. These trials resulted in one early period miscarriage and one healthy birth. The patient had never developed OHSS in any step of the treatments. To regulate her menstrual cycles, she took an estrogen (E2) and progesterone (P) combination. After the examination of the patient, other than a normal-sized uterus, small antral follicles were observed on the left ovarium, whereas the right ovarium was not monitored. In a previously taken hysterosalpingogram, the cavity and left tubal transition were normal; however, the right tuba was not monitored. Her body mass index (BMI) was 24 kg/m^2^. The basal characteristics of the patient are illustrated in Table 1. Her blood analysis resulted in white blood cells (WBC): 8.2, platelets: 307, hemoglobin (Hgb): 13.6, and hematocrit (Hct): 40. On the third day of menstruation (E2+P replacement triggered menstrual cycle), ovarian stimulation was started with 300 IU hMG (Menogon 75 Ferring Turkey). When three dominant follicles reached 18 mm in diameter, the final stage of oocyte maturation was triggered using 5000 IU urinary hCG (Pregnyl amp, MSD, Turkey). To avoid OHSS, the patient was medicated with cabergoline (Dostinex Pfizer Turkey) for 6 days after the trigger day. Transvaginal ultrasound-guided aspiration was performed 36 hours later. Two embryos, each grade 1, were transferred and three grade 1 embryos were frozen on the third day following intracytoplasmic sperm injection (ICSI). IVF outcomes are shown in Table 2. On the embryo transfer (ET) day, her WBC, Hgb, and Hct values were at normal levels. On the 9^th^ day following ET, she consulted our IVF center due to abdominal distention, swelling, and pain. Her ovaries were enlarged and abdominal fluid was observed on examination. Her blood analysis results were WBC: 22,400, Hct: 47.2, Hgb: 16.2, and beta-hCG: 61.7. Accordingly, she was accepted as having late OHSS and was hospitalized in a tertiary hospital. She stayed in hospital for 66 days. During this period, with 50,550 cc paracentesis fluid was aspirated; in total, 20% 100 cc human albumin and 0.9% NaCl was given 27 times, and hydroxyethyl starch was administered every day. The patient was discharged from hospital on the 13^th^ week of the pregnancy and she gave birth to a healthy girl via cesarean section.

A 26-year-old woman consulted our IVF center with symptoms of primary amenorrhea and primary infertility. She was married for 3 years. In her history, it was remarkable that she responded insufficiently to clomiphene citrate and had no menstruation after medroxyprogesterone acetate was administered; therefore, HMG was used until 225 units for controlled ovarian stimulation within an IUI plan. However, no response was received from the patient. When she was examined, it was determined that she had a small uterus and thin endometrium. Moreover, many small antral follicles were observed on both ovaries. Her basal characteristics are summarized in Table 1. Before starting treatment, a trial of GnRH analogue triggering was performed and 0.2 mg triptorelin (Gonapeptyl 0.1 Ferring, Turkey) was administered subcutaneously. After triggering, in the first and second hours, LH levels were 55 and 61 mIU/ML, respectively. Stimulation was started with 225 IU HMG (Menopur Ferring, Turkey) on the same day without waiting for menstruation. Follicle growth, as well as estradiol levels, were followed and gonadotropin doses were arranged according to the response. When at least three leading follicles (each >17 mm in diameter) were developed, oocyte maturation was triggered with 0.2 mg triptorelin. Thirty-two oocytes were retrieved 36 hours after the triptorelin injection. The IVF outcome of the patient is presented in Table 2. Following the first menstruation, she received hormone replacement therapy. One good quality embryo was transferred after the second menstruation using E2 hemihydrate (Estrofem 2 mg, Novanordisk Turkey) with a thawing protocol. This procedure resulted in pregnancy. At the time of writing, the patient was in her 13^th^ week of pregnancy.

## Discussion

In patients with HH, the aim of treatment is to achieve a single pregnancy through monofollicular development with stimulation and coitus or IUI. When ovulation induction and/or IUI fails or additional pathology (e.g. tubal obstruction or oligospermia) accompanies HH, treatment with ART is warranted. The first patient had severe OHSS, even though she had a single ovary. In the second case, response to stimulation was received over a long period and finally, an excessive ovarian response developed. However, the development of OHSS was prevented by performing an GnRH agonist stimulation test on the first day of treatment.

A fresh embryo was transferred to the first patient according to our IVF team’s decision, social reasons, and low risk of OHSS. Although early OHSS development did not occur, it developed with the beginning of the pregnancy as late OHSS with a long recovery duration lasting until the 13^th^ week of the pregnancy. Interestingly, she had a very serious OHSS clinic although she had a single small-sized ovary. Some 50.55 litres of acidic fluid was aspirated via repeated paracentesis. If the ET was cancelled, OHSS and its consequences would not occur. This case led us to reconsider the management of patients with HH with a hyper response to controlled ovarian stimulation.

Patients with HH are included in a heterogeneous population from the point of ovarian stimulation. Although the patients have the same clinical findings, their response to ovarian stimulation may be hyper, normal or poor. Both ovaries can be visualized as hypoplasic in ultrasonographic evaluation, hence, counting antral follicles is quite challenging. The prediction of the response to gonadotropins is challenging because the exact number of antral follicles cannot be identified and the levels of gonadotropins are below normal ranges^(7)^. AMH only reflects the growing follicular pool responsive to gonadotropins. Hence, conditions that cause a permanent or sustained interruption of gonadotropin release may lead to decreased AMH levels. Therefore, AMH is not a proper predictor of ovarian reserve in patients with HH, which may underestimate the exact ovarian reserve^(8)^. Determining the ovarian response before treatment is vital to provide acceptable rates of pregnancy with minimal adverse effects. Unfortunately, there is no reliable indicator for ovarian response in patients with HH except age^(7)^.

A high dose and long duration are needed during stimulation, which increases the risk of OHSS because ovaries are dormant and doubt of developing poor-response. It was stated that the ovarian stimulation duration could range from 12 to 54 days^(9)^. On the other hand, despite the application of a high dose, OHSS development is quite rare in patients with HH^(5,7,10,11,12)^. If OHSS occurs, the clinical condition can be life-threatening, as in our first case.

Kuroda et al.^(13)^ transferred a single frozen embryo with a freeze-all strategy to patients with HH to avoid the adverse effects of a fresh cycle on endometrial receptivity. They wanted to prevent a decreased response; therefore, the final oocyte was maturated with hCG, after considering the non-existence of OHSS in previous IVF cycles. They did not encounter any severe OHSS in their studies. Despite using the freeze-all strategy in his study, Kuroda et al.^(13)^ did not employ GnRH, which is the main trigger agent. The major reason was to eliminate the possibility of a decreased response with agonist triggering.

The cause of HH can be detected by looking at LH and FSH peak levels, after a GnRH agonist stimulation test^(14)^. No cut-off values for LH or FSH peak have been indicated. An increase in the peak equal or more than twofold is considered sufficient for maturation. This test would show a problem either with pituitary GnRH receptors or with GnRH production in the hypothalamus. If the condition is caused by pituitary GnRH receptors, LH and FSH levels will not increase, but if it is caused by GnRH, production gonadotropin levels will increase. Furthermore, even in women with GnRH receptor mutations, gonadotropin levels were detected to be increased like healthy controls in a previous study^(15)^. Women carrying GnRH receptor mutations were indicated to ovulate either spontaneously or in response to pulsatile GnRH administration via a portable pump^(15)^.

In our second case, it was planned to start the stimulation with high-dose gonadotropin because her BMI was high and no response was obtained despite long-term gonadotropin use in the previous IUI cycle. The possibility of triggering with GnRH agonist was considered due to the risk of OHSS. GnRH agonist triggering was performed knowing that the patient would respond because the LH increment was observed on the first day of the GnRH stimulation test. Thus, OHSS development was prevented and a healthy pregnancy was acquired in the second case. If LH response cannot be obtained after a GnRH stimulation test, combined use of alternative OHSS prevention methods is suggested (3).

Early or late OHSS can develop in patients with HH. Severe OHSS can be prevented with GnRH agonist triggering instead of hCG triggering in patients with HH with a hyper response. The use of a GnRH stimulation test will be very helpful for the selection of patients who will respond to GnRH agonist triggering before controlled ovarian stimulation cycles in patients with HH.

## Figures and Tables

**Table 1:**
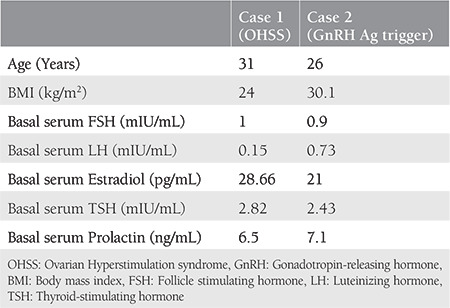
Clinical characteristic of two patients with hypogonadotropic hypogonadism

**Table 2:**
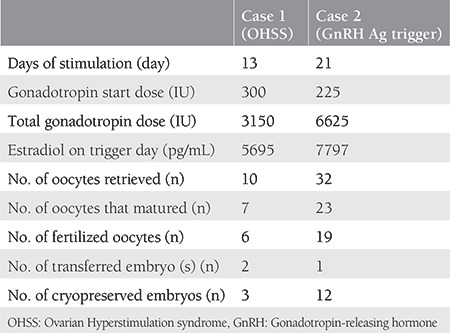
*In vitro* fertilization outcomes of two patients with hypogonadotropic hypogonadism
